
JOURNAL INFORMATION
==============================
NLM Title Abbreviation: Hemasphere
Journal ID: Hemasphere
NLM Journal ID: 101740619
Journal ID: HEM3

HemaSphere

EISSN: 2572-9241
Publisher: Wiley

ARTICLE INFORMATION
==============================
PMCID: PMC11499947
DOI: 10.1002/hem3.70043
Article ID: HEM370043
Article version: 1
Subjects: Issue Information, Issue Information

Issue Information

Electronic publication date: 2024 Oct 24
Publication date: 2024 Oct
Volume: 8
Issue: Suppl 2
Issue title: Abstracts of 13th International Symposium on Hodgkin Lymphoma
Electronic Location ID: e70043
Copyright: © 2024 The Authors. HemaSphere published by John Wiley & Sons Ltd. on behalf of European Hematology Association.
License: This is an open access article under the terms of the http://creativecommons.org/licenses/by/4.0/ License, which permits use, distribution and reproduction in any medium, provided the original work is properly cited.
License URL: https://creativecommons.org/licenses/by/4.0/

Figure count: 0
Table count: 0
Page count: 4

source-schema-version-number: 2.0
cover-date: October 2024
details-of-publishers-convertor: Converter:WILEY_ML3GV2_TO_JATSPMC version:6.4.9 mode:remove_FC converted:24.10.2024

==============================

### HemaSphere

Vol. 8 - Issue S2
October 2024

ISHL 13
COLOGNE GERMANY

OCTOBER 26-28, 2024

13th International Symposium on Hodgkin Lymphoma

### ABSTRACT BOOK

WILEY
hemaspherejournal.com
eha

### Aims and Scope

*HemaSphere* publishes results of highly relevant basic, translational, and clinical research in hematology. We are especially looking for strong studies reporting novel findings that are of high impact to the field of hematology.

Review articles and guideline articles provide clear overviews and discussions of new developments as well as recommendations for patient care.

*HemaSphere* is the premier hematology information resource, which is enhanced by the HemaTopics and HemaPolicy sections of the journal. These sections provide insightful discussions on all aspects related to hematology, including summaries of important articles, reports of new therapies, discussions on European policy, and other hematology news items. *HemaSphere* is guided by Editor in Chief Jan Cools and Deputy Editor in Chief Claire Harrison. The Editors and Editorial Board of *HemaSphere* are international research and clinical leaders, representing a diverse range of areas of hematologic expertise.

### Open Access Licensing and Copyright

*HemaSphere* is an Open Access journal: authors of accepted articles pay an article publication charge, and their articles are published under a Creative Commons license.

Certain funders mandate a particular type of CC license is used. All other authors can choose between the CC BY-NC and CC BY-NC-ND Creative Commons Licenses.

For more information about the licenses offered by this journal, please visit Wiley Author Services.

### Disclaimer

The Publisher, European Hematology Association, and Editors cannot be held responsible for any errors in or any consequences arising from the use of information contained in this journal. The views and opinions expressed do not necessarily reflect those of the Publisher, European Hematology Association, or Editors, neither does the publication of advertisements constitute any endorsement by the Publisher, European Hematology Association, Editors, or Authors of the products advertised. Wiley Open Access articles posted to repositories or websites are without warranty from Wiley of any kind, either express or implied, including, but not limited to, warranties of merchantability, fitness for a particular purpose, or non-infringement. To the fullest extent permitted by law Wiley disclaims all liability for any loss or damage arising out of, or in connection with, the use of or inability to use the content.

### Editorial Board

### Editor in Chief
**Professor Jan Cools**
*VIB-KU Leuven Center for Cancer Biology, Leuven, Belgium*

### Deputy Editor in Chief
**Professor Claire Harrison**
*Guy's and St Thomas' NHS Foundation Trust, London, UK*

### Associate Editors
Martin Dreyling, *Klinikum der Universität München, Germany*
Jeroen Eikenboom, *Leiden University Medical Centre, Leiden, The Netherlands*
Adele Fielding, *University of York, Hull York Medical School, York, UK*
Simon Hallam, *St. Bartholomew's Hospital, Barts Health NHS Trust, London, UK*
Robert Hills, *University of Oxford, UK*
Steffen Koschmieder, *University Medical Center RWTH, Aachen, Germany*
Marek Mraz, *CEITEC Masaryk University and University Hospital Brno, Brno, Czech Republic*
Paula Rodríguez, *Otero, Clínica Universidad de Navarra, Spain*
Juerg Schwaller, *University Hospital Basel, Switzerland*
Francesca Vinchi, *Iron Research Laboratory - New York Blood Center, & Weill Cornell Medicine Department of Pathology and Laboratory Medicine, New York, USA*

### Scientific Editors
Charles de Bock, *Children's Cancer Institute, Sydney, Australia*
Stephen Hibbs, *Queen Mary University of London, UK*
David Kent, *University of York, UK*
Edeghonghon Olayemi, *University of Ghana Medical School, Accra, Ghana*

### Editorial Manager, European Hematology Association
Jessica Mastrodomenico

### Editorial Board
Jeremy Abramson, *Massachusetts General Hospital Cancer Center, Boston, Massachusetts, USA*
Rafael Bejar, *University of San Diego, California, USA*
Marie Bene, *Centre Hospitalier Universitaire de Nantes, France*
Andrea Biondi, *University of Milano-Bicocca, Italy*
Jonathan Bond, *University College Dublin, Ireland*
Dominique Bonnet, *The Francis Crick Institute, London, UK*
Arndt Borkhardt, *University of Düsseldorf, Germany*
Dominique Bron, *Free University of Brussels, Belgium*
Christian Buske, *University of Ulm, Germany*
Suzanne Cannegieter, *Leiden University Medical Centre, The Netherlands*
Maria Domenica Capellini, *University of Milan, Italy*
Oliver Cornely, *University of Koeln, Germany*
Kim De Keersmaecker, *University of Leuven, Belgium*
Marianne De Montalembert, *Hôpital Universitaire Necker-Enfants Malades, Paris, France*
Michel Delforge, *Leuven Cancer Institute, Belgium*
Catherine Diefenbach, *Perlmutter Cancer Center, New York, USA*
Carlo Dufour, *IRCCS Istituto G. Gaslini, Genova, Italy*
Barbara Eichhorst, *University of Cologne, Germany*

Hermann Einsele, *University of Wurzbug, Germany*
Anna Falanga, *University of Milan Bicocca and Hospital Papa Giovanni XXIII, Bergamo, Italy*
Francesca Gay, *University of Turin, Italy*
Domenico Girelli, *Universita degli Studi di Verona, Italy*
Nicola Gökbuget, *University Hospital Frankfurt, Germany*
Kirsten Grønbæk, *Rigshospitalet, Copenhagen, Denmark*
Thomas Habermann, *Mayo Clinic, Rochester, Minnesota, USA*
Florian Heidel, *University Medical Center Greifswald, Germany*
Mark Hertzberg, *University of Sydney, Australia*
Brian Huntly, *University of Cambridge, UK*
Achille Iolascon, *University of Naples Federico II, Italy*
Shai Izraeli, *Schneider Children's Medical Center of Israel, Jerusalem, Israel*
Elaine Jaffe, *National Cancer Institute, Bethesda, Maryland, USA*
Efstathios Kastritis, *National and Kapodistrian University of Athens, Greece*
Marie Jose Kersten, *Academic Medical Center, Amsterdam, The Netherlands*
Jean-Jacques Kiladjian, *Hôpital Saint-Louis & Université Paris Diderot, France*
Daniela Krause, *University of Frankfurt, Germany*
Nicolaus Kröger, *University Hospital Hamburg-Eppendorf, Germany*
Ola Landgren, *Sylvester Comprehensive Cancer Center, University of Miami, Florida, USA*
Steven Lane, *QIMR Berghofer Medical Research Institute, Australia*
Maria-Victoria Mateos, *University of Salamanca, Spain*
Simon Mendez-Ferrer, *University of Cambridge, UK*
Thomas Mercher, *Gustave Roussy Institute, Villejuif, France*
Ann Mullally, *Harvard Medical School, Boston, Massachusetts, USA*
Stephen Mulligan, *Karolinska Institutet, Stockholm, Sweden*
Charlotte Niemeyer, *Albert-Ludwigs-Universität Freiburg, Germany*
Greg Nowakowski, *Mayo Clinic, Rochester, Minnesota, USA*
Esther Oliva, *Grande Ospedale Metropolitano Bianchi Melacrino Morelli, Calabria, Italy*
Jakob Passweg, *University of Basel, Switzerland*
Uwe Platzbecker, *University of Leipzig, Germany*
Marc Raaijmakers, *Erasmus Medical Center Cancer Institute, Rotterdam, The Netherlands*
David Rees, *University of London, UK*
Richard Rosenquist, *Karolinska Institutet, Stockholm, Sweden*
Davide Rossi, *Institute of Oncology Research, Bellinzona, Switzerland*
Simon Rule, *University Hospitals Plymouth NHS Trust, UK*
Rebekka Schneider, *Erasmus MC, Rotterdam, The Netherlands*
Radek Skoda, *University of Basel, Switzerland*
Kostas Stamatopoulos, *CERTH, Centre for Research and Technology Hellas, Thessaloniki, Greece*
Ulrich Steidl, *Albert Einstein College of Medicine/Montefiore Medical Center, New York, USA*
Anna Sureda, *Institut Català d'Oncologia, Barcelona, Spain*
Jan Trka, *University of Prague, Czech Republic*
